# Including the public perspective in health-related MCDA: ideas from the field of public opinion research and polling

**DOI:** 10.1186/s12962-018-0123-x

**Published:** 2018-11-09

**Authors:** Gillian K. SteelFisher

**Affiliations:** Harvard Opinion Research Program, Department of Health Policy and Management, Harvard T.H. Chan School of Public Health, 677 Huntington Avenue, Boston, MA 0211 USA

**Keywords:** Multi-criteria decisions analysis, MCDA, Public opinion, Poll

## Abstract

This commentary utilizes the lens of public opinion research in health to suggest ideas for best integrating public input into multi-criteria decisions analysis (MCDA) approaches. The field of public opinion research suggests: (1) there is frequently a distinction between public and expert views, even outside of situations where the public has direct experience; (2) representative samples are important to ensure findings reflect all segments of the relevant population; and (3) limiting cognitive burden in studies designed to elicit public preferences is essential for meaningful responses that represent the population’s views. Together these reflections suggest the need for considering new avenues for including public views in MCDA approaches where representative samples relying on well-designed questions are utilized more regularly.

## Background

There is increasing recognition of the value of incorporating the public perspective, including that of the general public, patients and family or consumers of services, in applications of multi-criteria decisions analysis (MCDA) to health care decisions [1]. As a result, substantial shares of institutions utilizing MCDA for Health Technology Assessment (HTA), for example, have integrated consumers into their processes, and the trend appears to be on the rise [2].

Despite increased interest in incorporating public views, there is not widespread agreement about how best to do this [1, 2]. There are an intense variety of approaches, with members of the public playing different roles—as stakeholder (i.e., the source of preferences), decision-makers (i.e., those that make choices between alternatives) or both—and no agreement or definitive guidance on the best applications for each role nor best practices within a given role. More fundamentally, there is not agreement on the justification for including consumers. While some suggest that individuals acting as decision-makers can ‘represent’ the public or patient perspective, others suggest that including members of the public merely broadens the range of perspectives in decision-making and enhances transparency [3]. With so many dimensions to clarify, there have been multiple calls for increased understanding and research about how best to incorporate the public perspective [1, 4].

As MCDA practitioners and researchers heed this call, it may be useful to consider input from other disciplines that have expertise in assessing public or patient opinion, including the field of public opinion research: the scholarly study of public views, which often aims to measure public preferences for policies as well as experiences with health concerns and services through surveys and polls. With a view from public opinion research as applied to health, this commentary focuses on three issues that may provide ideas for how public input is best integrated into MCDA approaches: the distinction between public and expert views; the role for representative samples in assessing public preferences; and the importance of limiting cognitive burden in studies designed to elicit public preferences.

First, the field of public opinion research provides ample evidence that the public and experts hold different perspectives on key issues—even outside of situations where the public may not have direct experience, such as having been a patient receiving care. The public may prioritize different concerns or may place value on different policy solutions [5, 6]. Further, public views may not align with other metrics and health indicators that experts use to assess the value of health technologies or the priorities for action [7]. This is not to say that broad public input is warranted on every decision. For example, there may be times when a topic is too technical or irrelevant to large swaths of the public, in which case a more selective sub-population may be needed to provide input. Good judgement on the part of the group designing the MCDA process, and perhaps best practice guidelines from the field more broadly, will be required. However, to support individuals or groups designing or guiding MCDA processes, evidence about the differences between public and expert sentiment reinforces the essential premise that it is worthwhile to include public views in MCDA processes when possible—even in cases when it is not self-evident, by virtue of their being patients, that consumers have a different viewpoint or a ‘unique perspective.’ Without including the public’s opinion, the MCDA process may result in decisions that directly conflict with public sentiment. This could lead to delays in implementation or rejection of MCDA results, particularly in circumstances where elected officials or others beholden to public sentiment have a say.

Second, the discipline of public opinion research recognizes the merits of gathering opinions from a statistically *representative* group of people through rigorous quantitative tools, which include a randomized sampling process from the relevant population. This is not to say that there is not a clear role for rigorous qualitative approaches that explicitly and purposefully include a non-representative sample. This can be important to provide richer insights into underlying values or provide opportunities for deliberation, particularly when a topic is too technical for broad public input, as suggested above. Rather, it is to say that public opinion research highlights the benefits of statistically representative samples in eliciting policy preferences, as this provides the opportunity to see what fraction of the entire public prefers a given policy. Some see the benefit of representative samples based on the parallels between polls and democratic processes or a moral imperative to include all views in policy-making [8]. There is also the very practical consideration that different sub-groups within the population have different views, as evidenced by current polls showing different policy preferences among people who identify with different political parties [9]. Thus, to state the near-obvious, non-representative samples used to rate or rank policy choices can overlook key perspectives and result in distorted outcomes. This is worrisome in the context of MCDA processes, where representative samples of consumers are not necessarily the norm, and non-representative samples are asked to provide quantitative ratings and rankings of various policies or their components. In these circumstances, it is important to consider which opinions—which segments of the public—are likely to be reflected in the results and which are not. Without explicit discussion of those absent from a given MCDA process, it can too easily be thought that any input from the public means input from all segments of the public.

Third, the discipline of public opinion research emphasizes that, in efforts to elucidate preferences, we should take seriously the cognitive burden that questions place on participants. Having survey questions that are easy to answer and will provide answers consistent with researchers’ intent is a fundamental principle of good survey design [10–12]. Only then can one interpret results meaningfully. In the context of MCDA, this provides challenges for approaches that utilize very complex survey questions or require very abstract thinking. Even if respondents *say* they can manage the cognitive challenge of such surveys, very substantial fractions—as much as 74% in one well-known study—provide responses that are not internally consistent [13]. Having such sizable fractions provide answers that are clearly at odds with researchers’ intent suggests that it is important to reconsider how respondents are being asked for their opinions. Moreover, it suggests that it is important to have experts with question-design skills on the team of those who develop MCDA approaches and tools. It may only be possible to design reasonable questions that tackle complex issues if there is sufficient question-writing skill, as well as time for robust pre-testing.

It is also worth noting that cognitive challenges in surveys can exacerbate the previously described limits of using non-representative samples in this context. While these MCDA techniques may use representative samples to start, it is standard practice in some approaches, such as analytic hierarchy process (AHP) study, to *remove from analysis* the responses of respondents who do not provide internally consistent answers. If large fractions are discarded, the final sample may not be representative at all and systematic distortions can occur. For example, it seems likely that less educated respondents will have a harder time answering cognitively complex questions consistently. If true, their responses are more likely to be removed, which will reduce the representativeness of the sample along the dimension of education and correlated attributes. More substantively, MCDA outcomes will give less voice to those with less access to education including those with lower incomes, those with lesser health status or racial/ethnic minorities.

## Conclusion

Together these reflections from the realm of public opinion research suggest the need for considering new avenues for including public views in MCDA where representative samples relying on well-designed questions are utilized more regularly. There are multiple options for integrating such public opinion data into MCDA processes, and the best point at which public opinion data are introduced would likely depend on the specifics of the topic or problem at hand as well as the proposed MCDA process. Two straightforward options include presenting data about public or consumer preferences to the decision-making group as another standard data input, alongside cost-effectiveness data, for example. Public opinion data could also be used in the steps of evaluation, such that decision-makers compare public preferences to MCDA outcomes in order to see how much they are aligned. In either option, the decision-makers will need to determine how much weight to give the public’s input, as they often do with other inputs. However, introducing high-quality public opinion data explicitly at these stages of MCDA processes ensures such a discussion occurs. Without it, public sentiment is either assumed or eclipsed.

Effective integration of high-quality public opinion data in one of these ways could have practical benefits beyond the direct benefits of including the public’s viewpoint in the process. If well-designed, high-quality data are included, it could support transparency by demonstrating to the public that their views were taken into account. In turn, this could have practical support for helping political leaders, who face electoral pressures, adopt MCDA approaches more frequently.

There are still many questions to explore about best practices for meaningfully integrating consumer views into MCDA processes both for individual efforts and as a means of developing best practices. While good judgement will always be needed on the part of those designing and implementing MCDA for a particular situation, the hope is that this commentary provides critical ideas from the field of public opinion research for consideration by such individuals as they push forward to meet the broader ambitions of improving the quality of decision-making and resultant public policy.

## References

[1] Thokala P, Devlin N, Marsh K, Baltussen R, Boysen M, Kalo Z (2016). Multiple criteria decision analysis for health care decision making—an introduction: report 1 of the ISPOR MCDA emerging good practices task force. Value Health.

[2] Hailey D, Werkö S, Bakri R, Cameron A, Göhlen B, Myles S (2012). Involvement of consumers in health technology assessment activities by INAHTA agencies. Int J Technol Assess Health Care.

[3] Daniels N, Sabin JE (2002). Accountability for reasonableness. Setting Limits Fairly: can we learn to share medical resources?.

[4] Ryan M, Scott D, Reeves C, Bate A, van Teijlingen E, Russell E (2001). Eliciting public preferences for healthcare: a systematic review of techniques. Health Technol Assess.

[5] SteelFisher G, Blendon R, Lasala-Blanco N (2015). Ebola in the United States—public reactions and implications. N Engl J Med.

[6] Funk C, Rainie L, Page D. Public and Scientists’ views on science and society. Pew Research Center, January 29, 2015. http://www.pewinternet.org/files/2015/01/PI_ScienceandSociety_Report_012915.pdf

[7] Abiola S, Gonzales R, Blendon R, Benson J (2011). Survey in sub-Saharan Africa shows substantial support for government efforts to improve health services. Health Aff.

[8] Glynn C, Herbst H, O’Keefe G, Shapiro R (1999). Public Opinion.

[9] Blendon R, Benson J, Casey L (2016). Health care in the 2016 election—a view through voters’ polarized lenses. N Engl J Med.

[10] Krosnick J, Presser S, Marsden P, Wright J (2010). Question and questionnaire design. Handbook of survey research.

[11] Converse J, Presser S (1986). Survey questions.

[12] Fowler F (2005). Improving survey questions.

[13] Hummel J, Steuten L, Groothuis-Oudshoorn C, Mulder N, IJzerman M (2013). Preferences for colorectal cancer screening techniques and intention to attend: a multi-criteria decision analysis. Appl Health Econ Health Policy.

